# Inappropriate antibiotic utilization in hospitalized patients in Ethiopia: A systematic review and meta-analysis

**DOI:** 10.1016/j.rcsop.2026.100809

**Published:** 2026-06-15

**Authors:** Tekletsadik Tekleslassie Alemayehu, Eskedar Dires Gebremeskel, Bezawit Dereje Tilahun, Tadele Mesfin Demelash, Tesfaye Birhanu Abebe, Seblewengel Hagos Tadesse, Gemechis Mekonnen Aga, Loza Getachew Chane, Mulat Alemu Beshada, Surafel Zimarge Abeje, Lydia Mekonen Geremew, Gelgela Gadisa Atomsa, Mastewal Mulugeta Wakero, Abaynesh Fentahun Bekalu, Gebremariam Wulie Geremew

**Affiliations:** a Department of Social and Administrative Pharmacy, School of Pharmacy, College of Medicine and Health Sciences, University of Gondar, Gondar, Ethiopia; b Department of Clinical Pharmacy, School of Pharmacy, College of Medicine and Health Sciences, University of Gondar, Gondar, Ethiopia; c School of Medicine, College of Health Science, Salale University, Fitche, Ethiopia; d School of Medicine, College of Medicine and Health Sciences, University of Gondar, Gondar, Ethiopia; e School of Medicine, Asrat Woldeyes Health Science Campus, Debre Berhan University, Debre Berhan, Ethiopia

**Keywords:** Inappropriate prescribing, Antibiotic use, Inpatients, Systematic review, Meta-analysis, Ethiopia

## Abstract

**Background:**

Inappropriate antibiotic use is a major driver of antimicrobial resistance and adverse clinical outcomes. Evidence on its pooled prevalence and determinants among hospitalized patients in Ethiopia remains scarce. Therefore, this study aimed to estimate the pooled prevalence of inappropriate antibiotic use and associated factors among hospitalized patients in Ethiopia.

**Methods:**

This systematic review and meta-analysis was reported in accordance with the PRISMA statement and registered in PROSPERO (CRD420261379594). A comprehensive literature search was conducted from December 5 and December 19, 2025. Electronic databases including PubMed/MEDLINE, Embase, ScienceDirect, Scopus, and PsycINFO were systematically searched. In addition, supplementary platforms and grey literature sources including Google Scholar, ResearchGate, and HINARI were explored to identify relevant studies. Data were extracted using Microsoft Excel and analyzed using Stata version 14.0. A random-effects model was employed to estimate the pooled prevalence, and heterogeneity was assessed using the I^2^ statistic. Subgroup analysis, sensitivity analysis, and assessment of publication bias were also performed.

**Results:**

Twelve primary studies were included. The pooled prevalence of inappropriate antibiotic use among hospitalized patients in Ethiopia was estimated at 40.50% (95% CI: 24.88–56.16). However, this estimate should be interpreted with caution due to the very high heterogeneity observed across studies (I^2^ = 99.5%). Inappropriate indication (26.2%) and incorrect duration of therapy (24.65%) were the most common forms of inappropriateness. Comorbid conditions (OR = 3.46; 95% CI: 2.79–4.28) and multiple medication co-prescription (OR = 2.98; 95% CI: 2.31–3.84) were significantly associated. The pooled prevalence of antibiotic utilization among hospitalized patients was 59.42% (95% CI: 48.76–70.07). Empirical therapy accounted for 93.26% of prescriptions, while culture and sensitivity testing was performed in only 17.7% of cases. The mean number of antibiotics prescribed per patient was 1.95. Metronidazole, cephalosporin, and penicillin were the most commonly prescribed antibiotics.

**Conclusion:**

Inappropriate antibiotic use among hospitalized patients in Ethiopia appears to be high and may be associated with empirical prescribing, limited diagnostic confirmation, comorbidities, and polypharmacy. Strengthening antimicrobial stewardship, and adherence to treatment guidelines is urgently needed.

## 1 Background

The burden of infectious diseases remains high, leading to extensive use of antibiotics.^1^, ^2^ Antibiotics are among the most frequently prescribed therapeutic agents in hospital settings and play a vital role in reducing morbidity and mortality caused by bacterial infections.^3^, ^4^ However, their widespread and often inappropriate use has become a major global public health concern.^5^ Hospitalized patients, in particular, are frequently exposed to broad-spectrum antibiotics, often prescribed empirically without microbiological confirmation. Studies reported high rates of inappropriate antibiotic use, particularly involving commonly prescribed agents such as ceftriaxone.^6^, ^7^ Globally, about 30% of antibiotics are used inappropriately, rising to approximately 36% in low- and middle-income countries.^8^ Moreover, a study by Boltena, M. T., et al. reported that the pooled prevalence of antibiotic use among hospitalized patients in sub-Saharan Africa was higher than from Europe and USA.^9^ This difference is linked to limited diagnostic capacity, weak regulatory systems, and inadequate antimicrobial stewardship programs.^10^ These inappropriate practices include unnecessary initiation of antibiotics, prolonged duration of therapy, incorrect dosing, and failure to adjust treatment based on clinical response or laboratory findings.^11^, ^12^

A previous systematic review conducted among the general population in Ethiopia reported a pooled prevalence of inappropriate antibiotic use of 49.2%.^1^ However, existing reviews have largely focused on estimating prevalence, with limited attention given to factors associated with inappropriate antibiotic use. In addition, variations in the definition and measurement of inappropriate use across studies have limited the comparability of findings. Despite a growing body of primary studies reporting inappropriate antibiotic use among hospitalized patients in Ethiopia, systematic reviews and meta-analyses remain scarce, and existing findings vary widely across regions, healthcare settings, and study populations. For instance, a study conducted in the Oromia region reported that 8.5% of antibiotics were used inappropriately,^13^ whereas a study from the Amhara region found a markedly higher level of inappropriate use, with 91.4% of antibiotics utilized incorrectly.^14^ Individual studies also revealed that comorbidities, polypharmacy, empirical treatment, and socio-demographic factors were associated with inappropriate antibiotic use.^11^, ^13^, ^14^ Reported prevalence rates also differ widely, reflecting inconsistencies in prescribing practices, and variations in study methodologies. This heterogeneity makes it difficult for policymakers, clinicians, and public health authorities to draw reliable national-level conclusions or design targeted interventions. Moreover, most individual studies are limited by small sample sizes, single-center designs, and lack of generalizability.

Inappropriate antibiotic utilization contributes significantly to antimicrobial resistance, reducing the effectiveness of available treatments and increasing the risk of treatment failure, prolonged illness, and mortality.^15^, ^16^ It also exposes patients to unnecessary adverse drug reactions, disrupts normal microbial flora leading to secondary infections, wastes of limited healthcare resources and increases healthcare costs through longer hospital stays and the need for more expensive second-line therapies.^17^, ^18^ To mitigate the problem antimicrobial stewardship programs are increasingly promoted in Ethiopia, however, their implementation remains limited,^19^ and evidence-based decision-making is constrained by the absence of pooled national estimates.

Given the increasing use of antibiotics and the potential consequences of inappropriate prescribing, to date, no comprehensive systematic review and meta-analysis has explicitly addressed the pooled prevalence of inappropriate antibiotic use and associated factors among hospitalized patients in Ethiopia. The absence of such evidence limits the ability of policymakers, clinicians, and antimicrobial stewardship programs to make informed, evidence-based decisions. Moreover, understanding the extent of heterogeneity and identifying potential sources of variation across studies are essential for targeted interventions. Therefore, conducting a systematic review and meta-analysis is necessary to generate a robust pooled estimate, improve the precision of existing evidence, and provide a comprehensive overview of inappropriate antibiotic use in Ethiopian hospital settings. Therefore, the aim of this study was to estimate the pooled prevalence of prevalence of inappropriate antibiotic use (IAU) and to identify associated factors among hospitalized patients in Ethiopia. The findings of this study will support national efforts to strengthen antimicrobial stewardship, inform clinical practice, and contribute to strategies aimed at combating antimicrobial resistance.

## 2 Method

### 2.1 Study protocol

The protocol for this systematic review and meta-analysis was prospectively registered in the International Prospective Register of Systematic Reviews (PROSPERO) under the identification number CRD420261379594 and is available at https://www.crd.york.ac.uk/PROSPERO/view/CRD420261379594. The review was reported in accordance with the Preferred Reporting Items for Systematic Reviews and Meta-Analyses (PRISMA) checklist, encompassing systematic literature searching, study selection, data extraction, and synthesis of findings^20^ (Supplementary File 1).

### 2.2 Search strategy

Relevant studies were systematically searched between December 5 and December 19, 2025, and included all articles published online up to the date of data collection. A comprehensive literature search was conducted using multiple electronic bibliographic databases and supplementary sources. The primary databases searched included PubMed/MEDLINE, Embase, ScienceDirect, Scopus, and PsycINFO. However, during the conduct of the review, the search strategy was expanded to enhance comprehensiveness and reduce publication bias. Accordingly, in addition to primary databases, additional searches were performed through supplementary platforms and sources including Google Scholar, ResearchGate, and HINARI to identify potentially relevant studies and grey literature. For Google Scholar, the first 125 results sorted by relevance were screened. Furthermore, reference list screening (snowballing) of included studies was performed, and attempts were made to contact corresponding authors when necessary to obtain additional or missing data. The search strategy employed a combination of key terms such as “prevalence, “magnitude, “pharmacoepidemiology”, “burden”, “inappropriate antibiotic use”, “inappropriate prescribing”, “irrational antibiotic use”, “antibiotic misuse”, “Anti-Bacterial Agents”, antibiotics“, antimicrobial, “associated factors, ““determinant, ““predictors, “ and “Ethiopia, “ using Boolean operators (“AND” and “OR”) as appropriate. The full search strategy used for each database and all supplementary sources is provided in Appendix 1.

### 2.3 Study selection

After identifying relevant articles through database searches, duplicate records were removed and the remaining studies were imported into EndNote X20. Four independent reviewers (TTA, GWG, SHT and EDG) conducted the screening process. Initially, titles and abstracts were screened to assess eligibility. Subsequently, full-text articles were independently evaluated against the predefined inclusion criteria. Any discrepancies among reviewers were resolved through discussion and consensus prior to data analysis.

### 2.4 Eligibility criteria

#### 2.4.1 Inclusion criteria

Observational studies, including original research articles, theses, and conference abstracts, published in English at any time, were eligible for inclusion if they assessed antibiotic utilization among hospitalized patients of any age group (pediatric, adult, or geriatric) admitted to any hospital ward for the management of infectious diseases. Fortunately, all studies included in this review were full-text, peer-reviewed publications; no conference abstracts were included. In line with the PECO framework, the population comprised hospitalized patients in Ethiopia, the exposure was inappropriate antibiotic utilization, and outcomes were defined as the prevalence, patterns and determinants of inappropriate antibiotic use. Inappropriate antibiotic use was defined, according to the criteria reported in each included study, as any deviation from national or international clinical guidelines or expert consensus recommendations.

#### 2.4.2 Exclusion criteria

Based on consensus among the authors, studies were excluded if they did not report the prevalence of inappropriate antibiotic use and/or associated factors, but merely described patterns or characteristics of inappropriate use. Articles that evaluated interventions without reporting baseline prevalence data prior to the intervention were also excluded. In addition, studies with incomplete or missing data were excluded when the required information could not be obtained from the authors. When uncertainty arose regarding study eligibility, the final decision was reached through discussion with three additional reviewers (BDT, TMD, and TBA).

#### 2.4.3 Data extraction

Data extraction was conducted using a predesigned and standardized Microsoft Excel form. Following article selection and eligibility confirmation, three independent reviewers (TTA, GWG, SHT, and EDG) extracted relevant data from the included studies. The extracted variables included the first author's name, year of publication, study region, study design, type of infectious disease, study population, data source, sample size, and commonly prescribed antibiotics. Information related to the outcomes of interest namely the prevalence and types of inappropriate antibiotic use (including inappropriate indication, dosage, duration, and frequency) was also collected. In addition, data on associated factors and effect estimates, including odds ratios with corresponding 95% confidence intervals, were extracted where available provided in (Supplementary Data). Any discrepancies identified during the extraction process were resolved through discussion and consensus among the reviewers. To ensure data accuracy and consistency, the extracted data were cross-checked by additional authors. Inter-rater reliability was assessed using Cohen's kappa statistic to evaluate the level of agreement among reviewers. Furthermore, a sensitivity analysis was performed to assess the robustness and stability of the pooled estimates generated in the meta-analysis.

#### 2.4.4 Outcome measurements

The primary outcomes of this systematic review and meta-analysis as registered in PROSPERO were the pooled prevalence of inappropriate antibiotic use among hospitalized patients in Ethiopia and the factors associated with inappropriate antibiotic use, reported as odds ratios. In addition to these pre-specified outcomes, several secondary and descriptive variables were extracted to provide a more comprehensive understanding of inappropriate antibiotic use patterns. These included types of inappropriate antibiotic use, including inappropriate indication, dose, duration, frequency, contraindications, and drug interactions; pooled prevalence of antibiotic use among hospitalized patients; proportion of empirical antibiotic therapy; proportion of culture and sensitivity testing; mean number of antibiotics prescribed per patient; most commonly prescribed antibiotics; and indications for antibiotic use.

#### 2.4.5 Quality assessment

The methodological quality of the included studies was assessed using the Joanna Briggs Institute (JBI) Critical Appraisal Checklist for Analytical Cross-Sectional Studies.^21^ This tool is appropriate for evaluating key domains such as sample selection, study setting, measurement validity, confounding factors, and appropriateness of statistical analysis. Each criterion in the checklist was rated as “yes,” “no,” “unclear,” or “not applicable.” For scoring purposes, a response of “yes” was assigned 1 point, while “no, and not applicable” 0-point “unclear,” were assigned 0.5 points. Consequently, the maximum attainable score was 8 points. Studies scoring ≥4 were considered moderate to high quality based on commonly used thresholds in previous systematic reviews utilizing the JBI tool. This cut-off was pre-specified before conducting the appraisal. Study quality assessment was primarily used to inform interpretation of findings and evaluate methodological rigor and was considered during sensitivity analyses; no studies were excluded solely on the basis of quality score, and no separate sensitivity analysis by study quality was performed. Each author independently assessed the quality of the eligible studies. Any discrepancies between reviewers' assessments were resolved by calculating the mean of the assigned scores. The quality assessment was completed on December 25, 2025.

### 2.5 Statistical procedure

Statistical analyses were performed using STATA version 14.0. The results of the included studies were synthesized using text, tables, and forest plots. Pooled estimates of inappropriate antibiotic use were calculated using the DerSimonian and Laird random-effects model. For each study, the standard error of prevalence was derived from the binomial distribution. To evaluate potential publication bias, we employed two complementary methods. First, funnel plots were visually inspected to assess the symmetry of study estimates. Second, formal statistical tests, including Egger's regression and Begg's rank correlation tests, were conducted at a 5% significance level. When evidence of publication bias was detected, the nonparametric “trim and fill” method developed by Duval and Tweedie was applied to estimate the number and impact of potentially missing studies and to adjust the pooled effect accordingly.

### 2.6 Heterogeneity assessment

Heterogeneity between articles was assessed by considering the I^2^ inconsistency statistic. Significant levels of heterogeneity were considered present when the I^2^ estimate was greater than or equal to 70%. Additionally, if we found evidence of heterogeneity during analysis, we used a sensitivity analysis to pinpoint its potential cause. We applied a leave-one-out sensitivity analysis to determine the potential cause of heterogeneity in the pooled prevalence of inappropriate antibiotic use. This was accomplished by systematically eliminating one author or one article.

### 2.7 Subgroup analyses

Subgroup analyses are useful for examining between-group differences or determining how a given group's characteristics affect the prediction of the pooled prevalence and the cause of heterogeneity across studies. In this study, the prevalence of inappropriate antibiotic use was examined by subgrouping the regions where the study was conducted, antibiotic appropriateness identification databases, number of databases used (single versus multiple), study design, and study population. The prevalence of inappropriate antibiotic use is reported as percentages with 95% confidence intervals.

## 3 Result

### 3.1 Article search results

**Fig. 1:**
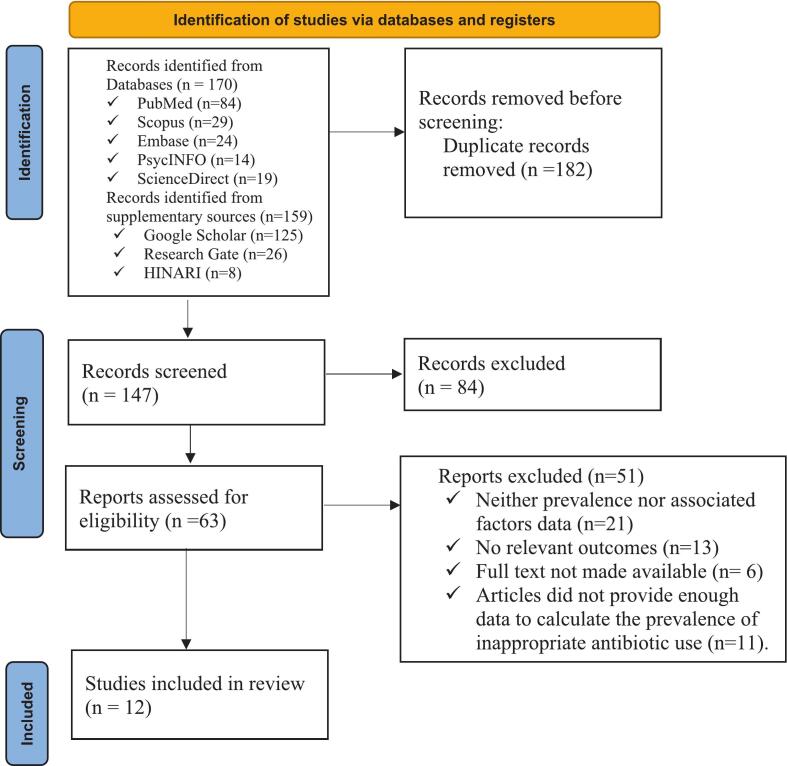
PRISMA 2020 flow diagram for systematic reviews.

The literature search yielded 329 records from electronic databases and manual searching. After removal of 182 duplicates, 147 articles remained for title and abstract screening, of which 84 were excluded. The full texts of 63 articles were assessed for eligibility, and 51 were excluded for predefined reasons (Supplementary file 2). Ultimately, 12 studies were included in the systematic review, and meta-analysis. The study selection process is summarized in the PRISMA flow diagram (Fig. 1).

### 3.2 General characteristics of the included studies

Twelve primary articles, comprising 5539 individuals, were included in the final systematic review and meta-analysis on the prevalence of inappropriate antibiotic use and determinants among hospitalized patients in Ethiopia. The included articles were published between 2012 and 2025. Geographically, the articles were obtained from two regions and one city administration (Addis Ababa). Half of the included studies employed a prospective cross-sectional design, while two studies used a retrospective cross-sectional design. The remaining four studies did not specify whether the cross-sectional design was prospective or retrospective and were reported simply as cross-sectional studies. Approximately*,* more than half of the included articles assessed inappropriate antibiotic use in terms of inappropriate duration, dose, indication, and frequency, whereas one article additionally assessed drug interactions and the use of contraindicated antibiotics. Eight different identification databases were used to detect inappropriate antibiotic use, with only two articles utilizing more than two identification databases. The national standard treatment guideline (NSTG) was used in four articles (33.3%), followed by the WHO guidelines in three articles (25%), and the RAND-modified Delphi method in two articles (16.7%) (Table 1).

**Table 1:** Characteristics of studies included in the systematic review and meta-analysis of inappropriate antibiotic utilization among hospitalized patients in Ethiopia (*N* = 12).

Authors	Publication year	Study design	Region	Sample size	Commonly prescribed antibiotics	Disease condition studied	Prevalence of inappropriate antibiotic use (%)
Tilahun et al.,	2023	PCS study	Addis Ababa	206	Ceftriaxone, vancomycin, and metronidazole	Pneumonia, meningitis, sepsis and septic shock, Brain abscess	24.8
Zeleke et al.,	2025	CS study	Amhara	412	Ceftriaxone, metronidazole, Cloxacillin, Ciprofloxacin and, Penicillin	Large bowel obstruction, Appendicitis, Fracture, and surgical site infection	64.5
Alekaw et al.,	2022	CS study	Amhara	279	Cephalosporin, penicillin's Metronidazole, and vancomycin	Pneumonia, sepsis and meningitis	30.8
Habteweld et al.,	2023	PCS study	Amhara	204	Cloxacillin, ciprofloxacin, ceftriaxone, Azithromycin, and metronidazole	Surgical site infection	77.9
Mama et al.	2020	CS study	Oromia	471	Cephalosporin, penicillin Metronidazole, and vancomycin	Genitourinary infection, Skin and soft tissue infection, Preoperative prophylaxis, Respiratory tract infection, Gastrointestinal infection sepsis, and CNS infection	30.1
Barghouthi Achalu and Mensa	2017	PCS study	Oromia	323	Crystalline penicillin, Cotrimoxazole, Macrolide, Cephalosporin, Chloramphenicol, Quinolones, Aminoglycosides, and Tetracycline's	Pneumonia, Acute gastro enteritis, Malnutrition, malaria, meningitis and sepsis	10.1
Agalu and Mekonnen,	2012	PCS study	Oromia	510	Cotrimoxazole Amoxicillin Mebendazole, Tetracycline Crystalline penicillin Cloxacillin Benzanthine penicillin Quinine	pneumonia, Acute Gastroenteritis,^20^ malaria	39.5
Gerina et al	2025	RCS study	Oromia	182	Penicillin, Macrolide, Cephalosporin, fluoroquinolones, Aminoglycosides, cotrimoxazole and Tetracycline	Pyogenic meningitis, tuberculosis, malaria, acute gastroenteritis and severe community-acquired pneumonia	8.5
Garedow et al., 2022	2022	PCS study	Oromia	402	Penicillin, Macrolide, Cephalosporin, Chloramphenicol, Quinolones/fluoroquinolones, Aminoglycosides, cotrimoxazole and Tetracycline	Meningitis, SAM, sepsis and lower respiratory tract infection	19.29
La Vecchia et al.,	2025	RCS study	Oromia	427	Ampicillin, Gentamicin, Ceftriaxone, Azithromycin, ceftriaxone, Azithromycin and Metronidazole	Pneumonia	43.9
Anteneh et al.,	2021	PCS study	Amhara	303	Cephalosporin, penicilins Metronidazole, Chloramphenicol and gentamicin	Pyogenic meningitis, tuberculosis, malaria, acute gastroenteritis and severe community-acquired pneumonia	91.4
Fentie et al.,	2021	CS study	National level	1820	Ampicillin, Ceftazidime, Gentamicin, Ciprofloxacin, Ceftriaxone, Meropenam, Azithromycin, ceftriaxone, Azithromycin, and Metronidazole	Pneumonia, sepsis, Active tuberculosis, and HIV	45.2

### 3.3 Quality of the included studies

The quality scores of the included studies, assessed using the JBI checklist, ranged from five to eight, indicating moderate to high methodological quality. A detailed overview of the JBI tool methodological quality assessment and ratings for each of the 12 studies included in this systematic review and meta-analysis can be found in Supplementary Table.

### 3.4 Study outcome measures

#### 3.4.1 Pooled prevalence of inappropriate antibiotic use among hospitalized patients in Ethiopia

**Fig. 2:**
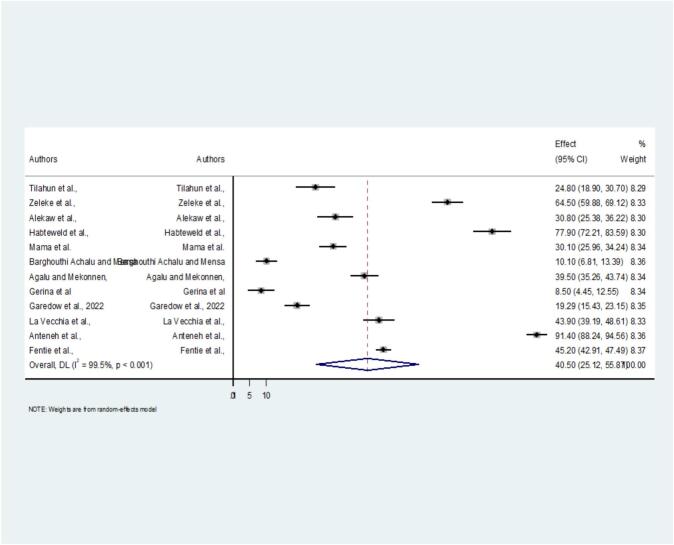
The pooled prevalence of inappropriate antibiotic utilization among hospitalized patients in Ethiopia. Note exp. (b) = Odds Ratio.

To determine the pooled prevalence of inappropriate antibiotic use among hospitalized patients in Ethiopia, a systematic review and meta-analysis were conducted using twelve published articles. The results revealed that the pooled prevalence of inappropriate antibiotic use among hospitalized patients in Ethiopia was 40.50% (95% CI: 24.88, 56.16) (Fig. 2). However, this estimate should be interpreted with caution due to the very high heterogeneity observed across studies (I^2^ = 99.5%). The included articles reported a wide range of inappropriate antibiotic use among hospitalized patients in Ethiopia, from 10.1%^22^ to 91.4%.^23^ When the type of inappropriate antibiotic use was considered, a substantial proportion of hospitalized patients 26.2% (95% CI: 13.28, 39.12) experienced inappropriate antibiotic use due to inappropriate indication, followed by an incorrect duration of treatment at 24.65% (95% CI: 14.52, 34.79). In contrast, inappropriate dosing and incorrect frequency of administration accounted for 12.75% (95% CI: 6.72, 18.78) and 11.89% (95% CI: 4.34, 19.44) of cases, respectively. While a single study reported that 12.9% and 20.8% of hospitalized patients experienced drug–drug interactions and contraindicated drug use, respectively^11^ (Table 2).

**Table 2:** Summary of types of inappropriate antibiotic use reported among hospitalized patients in Ethiopia.

Types of inappropriate antibiotic use	Number of study reported	Prevalence (95% CI)	Heterogeneity	
			I2 (%)	p- value
Inappropriate duration	8	24.65% (14.52, 34.79)	97.4	0.000
Incorrect dose	6	12.75% (6.72, 18.78)	90.4	0.000
Incorrect indication	7	26.2% (13.28, 39.12)	97.9	0.000
Inappropriate frequency	5	11.89% (4.34, 19.44)	93.2	0.000
Drug interactions	1	12.9%	–	–
Contraindications	1	20.8%	–	–

#### 3.4.2 Factors associated with inappropriate antibiotic use among hospitalized patients in Ethiopia

**Fig. 3:**
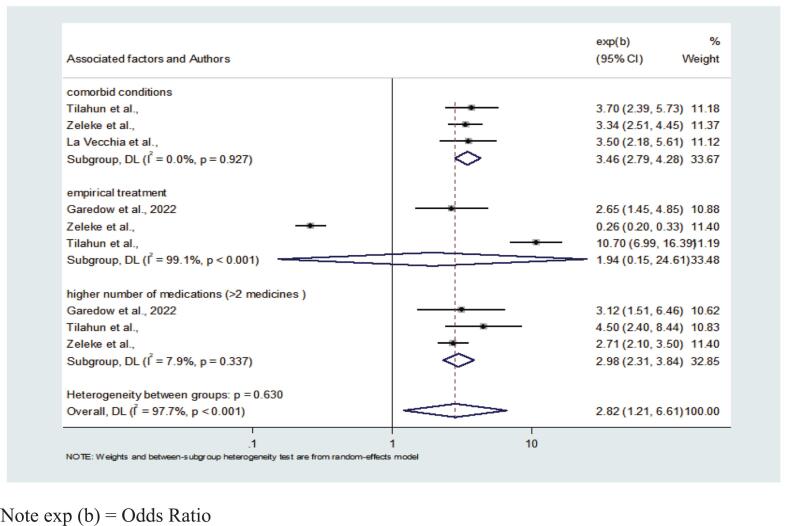
Factors associated with inappropriate antibiotic utilization among hospitalized patients in Ethiopia.

The pooled effect estimates were calculated using adjusted odds ratios (AORs) reported in the included studies. Comorbid conditions (OR = 3.46, 95%CI: 2.79, 4.28) and multiple medications co-prescription (OR = 2.98, 95%CI: 2.31, 3.84) were factors associated with inappropriate antibiotic use among hospitalized patients in Ethiopia (Fig. 3).

#### 3.4.3 The pooled prevalence of antibiotic utilization among hospitalized patients

The current study also assessed antibiotic utilization among hospitalized patients. The findings showed that the overall prevalence of antibiotic use among hospitalized patients in Ethiopia was 59.42% (95% CI: 48.76–70.07). The proportion of empirical antibiotic therapy was (93.26%; 95% CI: 86.30–100.2) derived from ten studies, whereas the proportion of culture and sensitivity testing was 17.7% (95% CI: 9.91–45.35) based on reports from six studies. The average number of antibiotics per patient was 1.95, based on eleven studies (Table 3).

**Table 3:** Antibiotic utilization among hospitalized patients in Ethiopia.

Types of inappropriate antibiotic use	Number of study reported	Prevalence (95% CI)	Heterogeneity	
			I2 (%)	p- value
Empirical treatment	10	93.26%(86.30, 100.0)	97.2	<0.001
Definitive treatment	6	6.74% (3.43, 37.02)	98.5	<0.001
Sensitivity testing	2	17.7% (9.91, 45.35)	98.5	<0.001
Overall antibiotic utilization		59.42% (48.76, 70.07)	99.3	<0.001

#### 3.4.4 The most commonly prescribed antibiotics

The analysis of antibiotic utilization among hospitalized patients showed that metronidazole, cephalosporin's, and penicillin's were the most commonly prescribed antibiotics.^12^, ^24^, ^25^, ^26^, ^27^, ^28^, ^29^, ^30^ Combination therapy was frequently used, with metronidazole often paired with a beta-lactam (penicillin or cephalosporin).^12^, ^24^, ^25^, ^27^, ^29^ Other frequently prescribed classes included Macrolides, Vancomycin, and broad-spectrum agents such as Quinolones and Aminoglycosides.^12^, ^24^, ^25^, ^29^ These findings also indicate a preference for broad-spectrum and combination regimens in the hospital setting.

#### 3.4.5 Indication for antibiotic use

The included studies involved patients with a wide range of infectious diseases admitted to medical, pediatric, surgical, gynecological, and emergency wards. The most commonly reported infections were pneumonia, meningitis, surgical site infections, urinary tract infections, sepsis, and soft tissue infections.^12^, ^24^, ^25^, ^26^, ^27^, ^28^, ^29^, ^30^, ^31^ Acute gastroenteritis and severe acute malnutrition were predominantly reported among pediatric patients.^12^, ^13^, ^24^, ^25^, ^30^ In contrast, large bowel obstruction, appendicitis, and neutropenic fever were less frequently reported conditions.^24^, ^30^, ^31^

### 3.5 Test of heterogeneity, publication bias, subgroups and sensitivity analysis

#### 3.5.1 Heterogeneity

The heterogeneity among the twelve included studies assessing inappropriate antibiotic use among hospitalized patients in Ethiopia was very high (I^2^ = 99.5%, *p* < 0.001). Given the extreme heterogeneity, a random-effects model was applied, and greater emphasis was placed on the range of reported estimates rather than the pooled effect alone.

#### 3.5.2 Publication bias

**Fig. 4:**
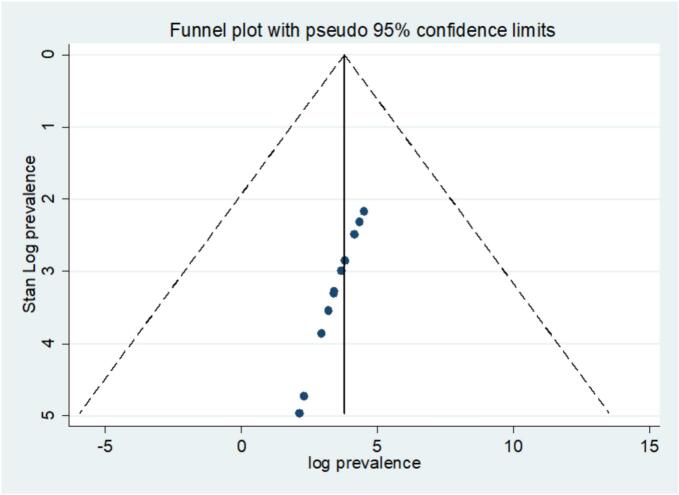
Funnel plot of logit event rates of inappropriate antibiotic utilization under a random-effects model plotted against standard error, used to visually explore potential small-study effects and publication bias.

To assess potential publication bias among the included studies, both visual inspection of the funnel plot and Egger's regression test were performed. The funnel plot appeared relatively symmetrical (Fig. 4), and Egger's test did not find strong evidence of small-study effects or publication bias (*p* = 0.787). However, these findings should be interpreted cautiously because the small number of included studies (Table 4).

**Table 4:** Egger's test for publication bias in studies estimating inappropriate antibiotic utilization among hospitalized patients in Ethiopia (N = 12).

Std_Eff	Coef.	Std. Err.	t	P > |t|	[95% Conf. Interval]
Slope	48.82915	27.07215	1.80	0.105	−12.4123 110.0706
Bias	−3.840732	13.78216	−0.28	0.787	−35.01815 27.33669

#### 3.5.3 Subgroup analysis

Subgroup analyses should be interpreted cautiously, as they were exploratory and hypothesis generating based on a small number of studies, wide confidence intervals, and substantial residual heterogeneity. Subgroup analyses were performed according to region, antibiotic appropriateness identification databases, number of databases used (single versus multiple), study design, and study population. The review demonstrated substantial variation in the pooled prevalence of inappropriate antibiotic use among hospitalized patients in Ethiopia across these subgroups. Subgroup analysis by study population showed that the highest prevalence of inappropriate antibiotic use was observed among adults (57.77%; 95% CI: 30.52–85.02), followed by pediatric patients (28.64%; 95% CI: 14.64–42.63) and studies including all age groups (26.89%; 95% CI: 9.08–62.86). Regional subgroup analysis indicated that the highest prevalence occurred in the Amhara region (66.63%; 95% CI: 46.78–86.49), followed by Addis Ababa and national-level studies (35.19%; 95% CI: 15.20–55.18). In contrast, the lowest prevalence was reported in the Oromia region (25.19%; 95% CI: 13.48–36.89). Regarding the identification databases used to assess inappropriate antibiotic use, studies applying the RAND-modified Delphi method reported the highest prevalence (78.00%; 95% CI: 51.64–104.37), followed by those using WHO guidelines (36.14%; 95% CI: 19.58–52.70). The lowest prevalence was observed in studies utilizing national standard treatment guidelines (22.02%; 95% CI: 7.35–36.70). Further subgroup analysis based on the number of databases used revealed that studies employing multiple databases reported a lower prevalence of inappropriate antibiotic use (24.03%; 95% CI: 12.92–35.13) compared with studies using a single database (43.59%; 95% CI: 26.06–61.11) (Table 5).

**Table 5:** Subgroup analysis of inappropriate antibiotic use among hospitalized patients in Ethiopia (N = 12).

Variable	Subgroup	Number of studies	Prevalence (95% CI)	I^2^%	*p* value
Region	Amhara	4	66.63% (46.78, 86.49)	98.4	0.000
	Oromia	6	25.19% (13.48, 36.89)	98.1	0.000
	Other*	2	35.19 (15.20, 55.18)	97.5	0.000
Study design	Retrospective cross- sectional	2	26.12% (8.5, 60.87)	99.2	0.000
	Prospective cross- sectional	6	43.83% (13.91, 73.74)	99.7	0.000
	Cross-sectional	4	42.95% (29.12, 56.78)	97.6	0.000
Database	RAND-modified Delph	2	78% (51.64, 96.11)	98.9	0.000
	NSTG	4	22.02% (7.35, 36.70)	98.2	0.000
	WHO Guideline	3	36.14% (19.58, 52.70)	99.5	0.000
	Other**	3	44.58% (7.23, 81.94)	98.9	0.000
Number of databases used	Single database	10	43.59% (26.06, 61.11)	76.4	0.000
	Multiple database	2	24.03% (12.92, 35.13)	99.5	0.000
Study population	Adult population	5	57.77% (30.52, 85.02)	99.5	0.000
	Pediatrics population	5	28.64% (14.64, 63.27)	98.0	0.000
	All age group	2	26.89% (9.08, 62.86)	99.5	0.000

Other* = Addis Ababa & national level study.

Other** = (Infectious Disease Society of America (IDSA), Medscape, Up-to-date version 21.6, Micromedex and American Society of Health-System Pharmacists.

#### 3.5.4 Sensitivity analysis

Sensitivity analysis was conducted in this systematic review and meta-analysis to evaluate the influence of individual studies on the pooled prevalence of inappropriate antibiotic use. The observation that all values remained within the expected 95% confidence intervals indicates that the exclusion of any single study did not substantially affect the overall pooled estimate. Additionally, attempts to remove one or more studies did not result in a significant reduction in heterogeneity. Consequently, twelve studies were retained for inclusion in the final systematic review and meta-analysis (Table 6).

**Table 6:** Sensitivity analysis of studies included in the meta-analysis of pooled prevalence of inappropriate antibiotic utilization among hospitalized patients in Ethiopia (N = 12).

Authors	Estimate prevalence (95% CI)	Heterogeneity	
		I2 (%)	p value
Tilahun et al.,	41.91 (25.62, 58.21)	99.5	<0.001
Zeleke et al.,	38.32 (22.12, 54.55)	99.5	<0.001
Alekaw et al.,	41.44 (24.95, 57.80)	99.5	<0.001
Habteweld et al.,	37.11 (21.34, 52.89)	99.5	<0.001
Mama et al.	41.44 (24.78, 58.11)	99.5	<0.001
Barghouthi Achalu and Mensa	43.38 (27.82, 58.72)	99.5	<0.001
Agalu and Mekonnen,	40.59 (23.92, 57.34)	99.5	<0.001
Gerina et al	43.41 (27.85, 59.15)	99.4	<0.001
Garedow et al., 2022	42.43 (26.12, 58.75)	99.5	<0.001
La Vecchia et al.,	40.26 (23.56, 56.81)	99.5	<0.001
Anteneh et al.,	35.83, 24.15, 47.60)	98.9	<0.001
Fentie et al.,	40.17 (21.88, 58.25)	99.5	<0.001

## 4 Discussion

### 4.1 Inappropriate antibiotic use among hospitalized patients in Ethiopia

This systematic review and meta-analysis provides comprehensive national-level evidence on the magnitude, patterns, and determinants of inappropriate antibiotic use among hospitalized patients in Ethiopia. Based on twelve eligible studies, the pooled prevalence of inappropriate antibiotic use was 40.50% (95% CI: 24.88, 56.16), however, this estimate should be interpreted with caution due to the very high heterogeneity observed across studies (I^2^ = 99.5%). The pooled estimate of inappropriate antibiotic use observed in the present study is comparable to a previous study conducted in Ethiopia, which reported a prevalence of 49%.^32^ This similarity may be due to several contextual and systemic factors that remain largely unchanged across study periods and settings. First, limited access to diagnostic and laboratory services in many Ethiopian healthcare facilities may be associated with greater reliance on empirical antibiotic therapy rather than culture- and sensitivity-guided prescribing.^33^ A finding from the present study, which found that 93.26% of antibiotic prescriptions were empirical, directly supports this explanation. Moreover, the lack of robust antimicrobial stewardship programs and monitoring mechanisms, inconsistent implementation of standard treatment guidelines, prescribing through trends, clinical experience and perceived patient expectations may explain why the pooled estimate in the present study aligns closely with findings from earlier research.^19^, ^34^ Furthermore, similarities in patient case mix, disease burden, and healthcare infrastructure across the study settings may also explain the comparable prevalence. Infectious diseases remain a leading cause of hospital admission in Ethiopia,^35^ creating high antibiotic demand and increasing the risk of misuse. Finally, inadequate in-service training and limited continuous professional development opportunities for prescribers may perpetuate long-standing prescribing behaviors.^36^

Similar patterns have been reported in China,^37^ Turkey^38^ and other low- and middle-income countries (LMICs),^39^ where inappropriate antibiotic use remains a persistent challenge. The comparability of high rates of inappropriate antibiotic use across different countries is not coincidental. Rather, it reflects a constellation of shared systemic failures that transcend individual national contexts, resulting in a common pattern of antibiotic misuse. Converging structural weaknesses such as fragmented and overburdened health systems, weak regulatory enforcement mechanisms, misaligned economic incentives, and limited access to reliable, affordable, and rapid diagnostic facilities constitute universal constraints that produce comparable outcomes. Additionally, sociocultural factors play a significant role, as antibiotics are widely perceived by patients and communities as powerful or universally effective medicines for treating a broad range of illnesses. This entrenched belief generates substantial patient demand and places considerable pressure on clinicians to prescribe antibiotics.^40^, ^41^ In such contexts, the prescription itself is often viewed as the tangible and expected outcome of a healthcare encounter.^42^ Consequently, prescribing becomes a low-risk, defensive strategy to avoid patient dissatisfaction, complaints, or perceptions of clinical negligence, particularly where alternative diagnostic justification is unavailable.^43^ Importantly, this cross-country comparability underscores that inappropriate antibiotic use is not an isolated or country-specific problem. Instead, it reframes the issue from one of localized practice deficiencies to a broader global health system failure. This perspective has important implications for intervention strategies; because the underlying drivers are largely shared, solutions proven effective in one LMIC setting are likely to be transferable and adaptable to others, supporting the development of universal antimicrobial stewardship frameworks.

When examining the specific types of inappropriate antibiotic use, inappropriate indication emerged as the most common problem, affecting more than one-quarter (26.2%) of hospitalized patients. This finding suggests that antibiotics are frequently prescribed without clear clinical justification, possibly for viral infections, non-infectious conditions, or as unnecessary prophylaxis.^44^ This practice may be associated with diagnostic uncertainty, fear of poor patient outcomes, high patient expectations, and limited access to laboratory confirmation of infections.45., 46 Inappropriate duration of antibiotic therapy was the second most frequent type in 24.65% hospitalized patients. This finding is supported by a previous study, which reported that patients admitted to intensive care units were exposed to inappropriate antibiotic therapy.^47^ This pattern reflects a tendency toward prolonged or inadequate treatment courses, both of which substantially contribute to the development of antimicrobial resistance, increased healthcare costs, and poorer patient outcomes.^48^ In contrast, inappropriate dosing (12.75%) and incorrect frequency of administration (11.89%) were less frequently reported but remain clinically significant. These issues may arise from inadequate dose adjustment for age, renal function, or disease severity, as well as insufficient monitoring of patients during therapy. Although drug–drug interactions and contraindicated antibiotic use were reported in only a single study, their presence is concerning, as they pose serious risks to patient safety. The limited reporting of these outcomes may reflect underassessment rather than true absence, highlighting a gap in current evaluation practices.

The wide range of reported prevalence across individual studies (10.1% to 91.4%), identified by this systematic review is similar to that reported in recent reviews. For example, one review reported that prevalence estimates for inappropriate antibiotic use ranged from 21% to 87.9%,^32^ whereas another reported a range of 22% to 76%.^38^ The wide variation in the prevalence of inappropriate antibiotic use among hospitalized patients in the current systematic review and meta-analysis is not a flaw in the research, but it tells us that the problem exists everywhere, but its severity is context-dependent. Such variability may reflect differences in study populations, methods used to assess antibiotic appropriateness, institutional prescribing policies, prescriber training, access to diagnostic services, and the availability and enforcement of treatment guidelines across regions and hospitals in Ethiopia. It underscores that the problem is not uniform but is instead a syndrome with varying severity across different parts of the health system.

However, when the prevalence estimates were pooled in a meta-analysis, substantial heterogeneity was observed between studies (I^2^ = 99.5%). This heterogeneity may reflect differences across studies in the databases used to assess antibiotic appropriateness, region, number of databases used (single versus multiple), study design, and study population. Such a high degree of heterogeneity reduces the reliability of the pooled estimate and suggests that it should not be interpreted as a single precise measure. Rather, the pooled prevalence represents an average across studies with diverse characteristics. Although subgroup and sensitivity analyses were conducted, considerable unexplained variability remained. Therefore, the findings from the subgroup analyses should be interpreted cautiously, as they were exploratory and hypothesis generating in nature and were based on a limited number of studies with wide confidence intervals and substantial residual heterogeneity. Subgroup analyses based on databases used to assess antibiotic appropriateness showed wide variation in pooled prevalence estimates, ranging from 22.02% those utilized NSTG to 78% in RAND-modified Delph. The variation could be due to databases such as NSTG are typically more conservative and context-specific, reflecting locally adapted recommendations that consider resource availability, prevailing disease patterns, and prescribing realities.^49^ As a result, assessments based on NSTG may be associated with classifying a larger proportion of prescriptions as appropriate, which could contribute to lower estimates of inappropriate antibiotic use. In contrast, the RAND-modified Delphi method applies a more stringent, expert-driven consensus framework that evaluates prescriptions against idealized evidence-based standards.^50^ This approach often incorporates broader clinical considerations, including optimal drug choice, dose, route, and duration, which is associated with a higher likelihood of classifying prescriptions as inappropriate. Furthermore, variability may also arise from differences in interpretation, expertise, and the level of clinical detail available in patient records.^51^ The lack of harmonization among appropriateness assessment tools limits direct comparability across studies and contributes to the observed heterogeneity in pooled estimates.

Ideally, information should be drawn from multiple sources and interpreted with caution. Consistent with this approach, studies employing multiple databases reported lower prevalence at 24.03% (95% CI: 12.92–35.13) compared with those using a single database at 43.59% (95% CI: 26.06, 61.11). This difference may be attributed to the complementary and cross-validating nature of multiple assessment databases. When multiple databases are employed, discrepancies in criteria, scope, and stringency across guidelines can be reconciled through triangulation, reducing the likelihood of misclassification and overestimation of inappropriate use. Broader assessment frameworks also allow contextual clinical justifications that might be overlooked when relying on a single, rigid standard. In contrast, studies using a single database are more vulnerable to the limitations and inherent biases of that specific tool, which may apply narrower or more stringent criteria and thereby classify a higher proportion of prescriptions as inappropriate. Additionally, single-database assessments may not adequately account for local prescribing realities, patient complexity, or resource constraints. Consequently, the use of multiple databases may provide more balanced, conservative, and clinically nuanced estimates of antibiotic appropriateness.^52^

Furthermore, the subgroup analysis revealed that inappropriateness antibiotic use differed based on the study population, with a higher prevalence in adult patients than in pediatrics patients, which revealed that the prevalence of inappropriateness antibiotic use among adult patients was (57.77% versus 28.64% in pediatric patients). This may be due to adult patients often present with multiple comorbidities, polypharmacy, and more complex clinical conditions, which increase diagnostic uncertainty and encourage empirical or broad-spectrum antibiotic use. Such complexity may increase the risk of inappropriate antibiotic selection, dosing, or duration.^53^, ^54^ In contrast, pediatric prescribing is generally more standardized, with clearer weight-based dosing protocols and well-established pediatric treatment guidelines, which may promote more appropriate antibiotic use.^55^, ^56^ Additionally, pediatric patients tend to receive closer clinical supervision, and prescribers may exercise greater caution because of concerns about drug safety and adverse effects in children.^57^ The relatively limited antibiotic options approved for pediatric use may also restrict inappropriate prescribing. Conversely, adult care settings often allow greater prescribing autonomy and access to a wider range of antibiotics, which may contribute to higher rates of misuse.

Finally, regional subgroup analysis indicated that the highest prevalence occurred in the Amhara region (66.63%), followed by Addis Ababa and national-level studies (35.19%). In contrast, the lowest prevalence was reported in the Oromia region (25.19%). This finding is in line with a previous study.^58^ This could be due to regional differences in healthcare infrastructure, prescribing practices, and implementation of antimicrobial stewardship interventions.^19^ Regional variations in patient case mix, disease burden, and healthcare utilization patterns may affect antibiotic prescribing practices. Differences in the availability and enforcement of standard treatment guidelines and antimicrobial stewardship programs may also contribute to the observed disparities. In addition, disparities in prescriber training opportunities, workload, and institutional oversight across regions may further account for the regional variation in inappropriate antibiotic use.

### 4.2 Factors associated with inappropriate antibiotic use among hospitalized patients in Ethiopia

The present study found that the presence of comorbid conditions was significantly associated with inappropriate antibiotic use among hospitalized patients in Ethiopia (OR = 3.46; 95% CI: 2.79–4.28). A study from other low- and middle-income countries (LMICs), was support this finding. This finding indicates that patients with one or more chronic illnesses were more than three times more likely to receive inappropriate antibiotic therapy compared with patients without comorbidities. A study from other low- and middle-income countries (LMICs), was support this finding.^59^ This association can be explained by several clinical and system-related factors. Patients with comorbid conditions such communicable and none-communicable disease often present with atypical clinical manifestations, making accurate diagnosis of infection more difficult.^60^ As a result, health professionals may initiate empirical antibiotics to avoid potential complications, thereby increasing the risk of inappropriate prescribing. Additionally, comorbidities are frequently associated with recurrent hospital admissions, prolonged hospital stays, and increased exposure to invasive procedures, all of which raise clinicians concern for hospital-acquired infections.^61^, ^62^ This often leads to defensive prescribing practices, including unnecessary initiation of antibiotics or prolonged treatment duration, even when bacterial infection is not confirmed. Furthermore, comorbid patients are more likely to have altered pharmacokinetics and pharmacodynamics, particularly in renal or hepatic impairment.^63^, ^64^ In the absence of routine dose adjustment or therapeutic drug monitoring, this may lead to inappropriate antibiotic selection, dosing, or duration.

The current study also revealed that patients receiving multiple medications were nearly three times more likely to receive inappropriate antibiotics (OR = 2.98; 95% CI: 2.31–3.84). This finding is consistent with previous studies that have identified polypharmacy as a major risk factor for irrational drug use in hospitalized patients.^65^ Polypharmacy complicates clinical decision-making and increases the risk of drug–drug interactions, therapeutic duplication, and dosing errors.^66^ In such situations, antibiotics are often added empirically without adequate review of existing medications or confirmation of infection. This problem is particularly pronounced in patients with chronic illnesses who are already on long-term medications. Moreover, polypharmacy increases the risk of adverse drug reactions, which may mimic infectious symptoms such as fever or elevated inflammatory markers.^67^ This can mislead clinicians into continuing or escalating antibiotic therapy unnecessarily.

### 4.3 Antibiotic utilization among hospitalized patients

The present analysis revealed that approximately 59% of hospitalized patients in Ethiopia received at least one antibiotic, indicating a high level of antibiotic exposure in inpatient settings. This finding is consistent with previous reports from low- and middle-income countries (LMICs), where antibiotic utilization rates 52% of patients.^68^ Similar prevalence rates have been documented in studies from china 50.3%,^69^ 47.7% and 42.1% across the globe^70^, ^71^ reflecting a widespread pattern of heavy antibiotic use. The high prevalence of antibiotic use in Ethiopian hospitals is driven by multiple interrelated factors, including the substantial burden of infectious diseases, inadequate access to diagnostic and microbiological testing, and clinical uncertainty in patient management.^72^, ^73^ These challenges often compel clinicians to rely on empirical therapy and adopt defensive prescribing practices to avoid potential adverse outcomes, thereby increasing overall antibiotic utilization. While high antibiotic use may reflect the genuine burden of infectious diseases, it also raises serious concerns regarding inappropriate prescribing, antimicrobial resistance, drug–drug interactions, prolonged hospital stays, and treatment failure and increased healthcare costs.^68^, ^74^ The mean number of antibiotics prescribed per patient in the present study was 1.95, which is broadly comparable with findings from India, where a mean of 2.95 antibiotics per patient has been reported.^75^ This alignment may be attributed to similarities in healthcare delivery systems in low- and middle-income countries, including a high burden of infectious diseases, limited access to definitive diagnostic facilities, and a tendency toward empirical antibiotic prescribing.

Notably, the overwhelming majority of prescriptions were empirical (93.26%) while culture and sensitivity testing was performed in fewer cases. This heavy reliance on empirical therapy likely reflects limited microbiology laboratory infrastructure, delayed test results, and cost constraints.^76^ While empirical treatment is often necessary in acute care settings, the lack of microbiological confirmation may perpetuate inappropriate antibiotic selection and duration, thereby increasing the risk of resistance development.^77^ The mean number of antibiotics prescribed per patient (1.95) further suggests frequent use of combination therapy, which may be justified in some clinical contexts but can also indicate irrational polypharmacy. Without appropriate justification, combination antibiotic therapy increases the risk of adverse effects, drug interactions, and unnecessary antimicrobial exposure.^72^

### 4.4 The most commonly prescribed antibiotics

The analysis of antibiotic utilization among hospitalized patients showed that metronidazole, cephalosporin's, and penicillin's were the most commonly prescribed antibiotics. Combination therapy was frequently used, with metronidazole often paired with a beta-lactam (penicillin or cephalosporin). Other frequently prescribed classes included Macrolides, Vancomycin, and broad-spectrum agents such as Quinolones and Aminoglycosides. These findings also indicate a preference for broad-spectrum and combination regimens in the hospital setting. This pattern aligns with several previous studies, which have reported similar trends in hospital antibiotic prescribing.^68^, ^78^ This alignment may be due to several factors. First, empirical prescribing is common in hospital settings, particularly where microbiological diagnostic capacity is limited, leading clinicians to favor broad-spectrum agents to cover a wide range of potential pathogens. Second, the severity and complexity of infections in hospitalized patients often necessitate combination therapy to ensure adequate coverage of both aerobic and anaerobic bacteria. Finally, concerns about antimicrobial resistance and previous treatment failures may further drive clinicians to use broader coverage to improve the likelihood of successful outcomes.

### 4.5 Indication for antibiotic use

The spectrum of infections reported in the included studies aligns with patterns observed in prior research, where pneumonia, urinary tract infections, sepsis, and surgical site infections are consistently among the most prevalent conditions in hospitalized patients across medical and surgical wards.^79^, ^80^ Similar to earlier studies, pediatric populations in these reports showed a higher burden of acute gastroenteritis and severe acute malnutrition, reflecting the vulnerability of children to infectious and nutrition-related conditions (81). However, less commonly reported conditions such as large bowel obstruction, appendicitis, and neutropenic fever were infrequently captured, which is consistent with other hospital-based studies that highlight these as relatively rare but clinically significant presentations (82). Overall, these findings underscore the broad range of infectious diseases affecting both adult and pediatric populations, while emphasizing the need for targeted interventions for the most frequent and high-risk conditions, corroborating evidence from both regional and global hospital-based surveillance studies.

### 4.6 Implications of the study

The findings of this systematic review and meta-analysis have important clinical, policy, and research implications for improving antibiotic use among hospitalized patients in Ethiopia. The high pooled prevalence of inappropriate antibiotic use (40.5%) indicates substantial gaps in rational prescribing practices and highlights an urgent need for strengthened antimicrobial stewardship interventions at hospital and national levels. Moreover, the predominance of inappropriate indication and treatment duration, along with extensive empirical prescribing and limited use of culture and sensitivity testing, highlights gaps in diagnostic capacity and guideline adherence. Strengthening laboratory services, promoting evidence-based prescribing, and ensuring consistent implementation of the NSTG are critical to improving antibiotic use. In addition to this, the association of inappropriate use with comorbidities and polypharmacy further emphasizes the importance of multidisciplinary care and routine medication review. Overall, these findings support the integration of standardized stewardship programs, continuous professional training, and regular prescription audits to optimize antibiotic use and mitigate the growing threat of antimicrobial resistance in Ethiopia. Finally, marked regional and methodological variations in inappropriate antibiotic use suggest inconsistencies in guideline implementation and assessment approaches. This finding highlights the need for harmonized national standards, wider dissemination and enforcement of the NSTG, and the adoption of standardized antibiotic appropriateness assessment tools across healthcare facilities.

## 5 Limitations of the study

This systematic review and meta-analysis has some limitations that should be considered when interpreting the findings. First, substantial heterogeneity was observed among the included studies (I^2^ = 99.5%), which may be attributed to variations in study design, study populations, regional distribution, and the criteria used to define inappropriate antibiotic use. Although subgroup and sensitivity analyses were performed to explore potential sources of heterogeneity, these analyses did not fully explain the observed variability. Second, the identification of inappropriate antibiotic use was not uniform across studies. Different assessment tools and guidelines including national standard treatment guidelines, WHO guidelines, and the RAND-modified Delphi method were applied, which may have resulted in inconsistent classification and affected the pooled prevalence estimates. In addition, most studies relied on a single identification database, potentially underestimating or overestimating the true magnitude of inappropriate antibiotic use. Third, the majority of the included studies employed cross-sectional designs, limiting the ability to establish causal relationships between identified determinants and inappropriate antibiotic use. Third, the assessment of publication bias using Egger's and Begg's tests should be interpreted with caution, as only 12 studies were included in the meta-analysis. With a limited number of studies, these statistical tests have low power to reliably detect publication bias, particularly in the presence of high heterogeneity. Furthermore, several studies did not clearly specify whether the cross-sectional design was prospective or retrospective, which might affect the assessment of data quality and temporal relationships. Finally, the geographical representation of the included studies was limited to two regions and one city administration, which may restrict the generalizability of the findings to other regions of Ethiopia with different healthcare infrastructures and prescribing practices.

## 6 Conclusion

This systematic review and meta-analysis suggests that inappropriate antibiotic use among hospitalized patients in Ethiopia remains an important public health concern. Although the included studies indicated a substantial burden of inappropriate antibiotic use, the findings should be interpreted cautiously due to considerable heterogeneity across studies, limited geographic representation, and variability in the definitions and assessment methods used. Inappropriate antibiotic use among hospitalized patients was primarily due to incorrect indications and treatment duration, while dosing and administration frequency errors were less common. Overall, antibiotic utilization among hospitalized patients in Ethiopia was high, with the majority of prescriptions initiated empirically. Comorbid conditions and multiple medications co-prescription were significant determinants of inappropriate antibiotic use. Nevertheless, the predominance of broad-spectrum and combination antibiotic regimens indicates a consistent prescribing pattern across hospital settings. Ultimately, this study provides a comprehensive synthesis of inappropriate antibiotic use among hospitalized patients in Ethiopia and offers important evidence to support the development of antimicrobial stewardship programs and inform national policies on the rational use of antibiotics.

## Human ethics and consent to participate

Not applicable.

## Clinical trial number

Not applicable.

## Availability of data and materials

All relevant data are available within the manuscript.

## CRediT authorship contribution statement

**Tekletsadik Tekleslassie Alemayehu:** Writing – original draft, Visualization, Validation, Supervision, Software, Resources, Project administration, Methodology, Formal analysis, Data curation, Conceptualization. **Eskedar Dires Gebremeskel:** Writing – review & editing, Writing – original draft, Methodology, Data curation, Conceptualization. **Bezawit Dereje Tilahun:** Writing – review & editing, Supervision, Methodology, Data curation. **Tadele Mesfin Demelash:** Writing – review & editing, Methodology, Data curation, Conceptualization. **Tesfaye Birhanu Abebe:** Writing – review & editing, Writing – original draft, Software, Methodology, Formal analysis, Data curation, Conceptualization. **Seblewengel Hagos Tadesse:** Writing – review & editing, Writing – original draft, Methodology, Data curation, Conceptualization. **Gebremariam Wulie Geremew:** Writing – review & editing, Visualization, Validation, Supervision, Software, Methodology, Data curation, Conceptualization.

## Ethical approval

Not applicable.

## Funding

The authors were not funded for this work.

## Declaration of competing interest

The authors declare that they have no competing interests.

## Appendix A Search Terms Used

The searches were conducted between December 5 and December 19, 2025. The same Boolean operators (“AND” and “OR”) and truncation symbol (“*”) were consistently applied across all databases and information sources to combine search concepts and retrieve variations of relevant terms. Searches were limited to primary studies published in the English language and conducted among human participants. The number of results screened is provided in Fig. 1.

The following search strategy was used in PubMed/MEDLINE.

(“Anti-Bacterial Agents”[Mesh] OR antibiotic*[tiab] OR antimicrobial*[tiab])

AND

(Inappropriate [tiab] OR irrational [tiab] OR improper [tiab] OR misuse [tiab] OR overuse[tiab] OR underuse[tiab] OR “inappropriate utilization”[tiab] OR “inappropriate use”[tiab])

AND

(Prevalence [tiab] OR occurrence [tiab] OR epidemiology [Subheading] OR pharmacoepidemiology [tiab] OR magnitude [tiab] OR burden [tiab])

AND

(Hospital*[tiab] OR inpatient*[tiab] OR “Hospitalized Patients”[Mesh])

AND

(Ethiopia [Mesh] OR Ethiopia [tiab])

Field tags used: [tiab], [mesh], [au].

The following search strategy was used in Embase.

(‘antibiotic agent’/exp. OR antibiotic*:ti,ab OR antimicrobial*:ti,ab)

AND

(Inappropriate:ti,ab OR irrational:ti,ab OR improper:ti,ab OR misuse:ti,ab OR overuse:ti,ab OR underuse:ti,ab OR ‘inappropriate utilization’:ti,ab OR ‘inappropriate use’:ti,ab)

AND

(Prevalence:ti,ab OR occurrence:ti,ab OR pharmacoepidemiology:ti,ab OR magnitude:ti,ab OR burden:ti,ab)

AND

(Hospital*:ti,ab OR inpatient*:ti,ab OR ‘hospitalized patient’/exp)

AND

(Ethiopia:ti,ab OR ‘Ethiopia’/exp)

Field tags used: Embase::ti,ab, /exp, /de.

*The following search strategy was used in ScienceDirect.*

(“antibiotic use” OR antibiotic* OR antimicrobial)

AND

(Inappropriate OR irrational OR improper OR misuse OR overuse OR underuse OR “inappropriate utilization” OR “inappropriate use”)

AND

(Prevalence OR occurrence OR burden OR magnitude OR epidemiology OR pharmacoepidemiology)

AND

(Hospital OR hospitalized OR inpatient)

AND

(Ethiopia)

Field tags used: Searches were usually written without tags.

*The following search strategy was used in HINARI.*

(Antibiotic OR antimicrobial)

AND

(Inappropriate OR irrational OR misuse OR overuse OR underuse OR “inappropriate utilization”)

AND

(Prevalence OR occurrence OR pharmacoepidemiology OR epidemiology OR burden)

AND

(Hospital* OR inpatient*)

AND

(Ethiopia)

Field tags used: HINARI is a portal that provides access to multiple publisher. Field tags in HINARI searching are platform-dependent.

*The following search strategy was used in* Scopus.

(Antibiotic* OR antimicrobial*.

OR “anti-bacterial agents”)

AND

(Inappropriate OR irrational OR improper OR misuse OR overuse OR underuse.

OR “inappropriate utilization” OR “inappropriate use”)

AND

(Prevalence OR occurrence OR burden OR magnitude OR epidemiology OR pharmacoepidemiology)

AND

(Hospital* OR inpatient* OR “hospitalized patients”)

AND

(Ethiopia)

Field tags used: TITLE-ABS-KEY.

*The following search strategy was used in PsycINFO.*

(DE “Antibiotics” OR antibiotic* OR antimicrobial*)

AND

(Inappropriate OR irrational OR misuse OR overuse OR underuse OR “inappropriate utilization”)

AND

(Prevalence OR occurrence OR epidemiology OR pharmacoepidemiology OR burden)

AND

(Hospital* OR inpatient*)

AND

(Ethiopia)

Field tags used.

DE = Descriptor/subject heading.

*The following search strategy was used in Google Scholar.*

Search string 1: “Inappropriate antibiotic use” hospitalized patients Ethiopia.

Search string 2: (Prevalence antibiotic OR antimicrobial inappropriate utilization Ethiopia hospital).

Search string 3: irrational antibiotic use inpatient Ethiopia.

Screening procedure: Results were sorted by relevance and the first 125 records were screened because relevance substantially decreased after the initial pages.

The following search strategy was used in ResearchGate.

Search terms used: “inappropriate antibiotic utilization” Ethiopia hospital

“Irrational antibiotic use” inpatient Ethiopia.

“Antibiotic misuse” hospitalized patients Ethiopia.

Screening procedure: Titles and abstracts were screened manually. Then duplicate studies already identified from bibliographic databases were removed. Potentially eligible full texts were assessed using the same predefined inclusion criteria.
