# Supplementary material for: Inappropriate antibiotic utilization in hospitalized patients in Ethiopia: A systematic review and meta-analysis
Source: Explor Res Clin Soc Pharm. 2026 Jun 15;23:100809. doi: 10.1016/j.rcsop.2026.100809 (PMC13320460; doi:10.1016/j.rcsop.2026.100809)
Supplement: Supplementary file 2 — Supplementary material 2 [file mmc2.docx]

**Table S2.** List of studies excluded at the full-text screening stage with reasons for exclusion.

| Reason for Exclusion | Number of studies (n) | Author, Year |
| --- | --- | --- |
| - Neither prevalence nor associated factors data | 21 | Anagaw et al., 2023, Birhanu et al., 2023, Getachew et al., 2013, Kasse et al., 2025, Yimenu et al., 2019, Abebe et al., 2024, Teni et al., 2017, Mengistu et al., 2020, Wubetu et al., 2018, Gobezie et al., 2025, Dereje et al., 2023, Sahilu et al., 2023, Sisay et al., 2020,………. |
| - No relevant outcomes | 13 | Sisay et al., 2020, Gashaw et al., 2018, Edessa et al., 2024, Lenjisa et al., 2014, Bilal et al., 2016, Desalegn, 2013, Hailesilase et al., 2024, Teklay et al., 2024, Yilma and Liben, 2020, Teklay and Yohannes, 2025……….. |
| - Full text not made available | 6 | Demoz et al., 2020, Sema et al., 2021, Anagaw et al., 2023, Tadesse et al., 2022, Dessu et al., 2021….. |
| - Articles did not provide enough data to calculate the prevalence of inappropriate antibiotic use | 11 | Abejew et al., 2024, Boltena et al., 2026, Gashaw et al., 2021, Abebe et al., 2024, Tsegay et al., 2025, Yehualaw et al., 2021…….. |
