# Supplementary material for: Inappropriate antibiotic utilization in hospitalized patients in Ethiopia: A systematic review and meta-analysis
Source: Explor Res Clin Soc Pharm. 2026 Jun 15;23:100809. doi: 10.1016/j.rcsop.2026.100809 (PMC13320460; doi:10.1016/j.rcsop.2026.100809)
Supplement: Supplementary file 3 — Supplementary material 3 [file mmc3.docx]

Table_S2 Quality assessment of articles included in inappropriate antibiotic utilization among hospitalized patients in Ethiopia.

| Author, year of  Publication | Q1 | Q2 | Q3 | Q4 | Q5 | Q6 | Q7 | Q8 | Total score |
| --- | --- | --- | --- | --- | --- | --- | --- | --- | --- |
| Tilahun et al., | Y | Y | Y | Y | Y | U | Y | Y | 8 |
| Zeleke et al., | Y | Y | Y | Y | Y | Y | Y | Y | 8 |
| Alekaw et al., | Y | Y | NA | Y | Y | Y | Y | NA | 6 |
| Habteweld et al., | Y | Y | Y | Y | Y | Y | Y | NA | 7 |
| Mama et al. | Y | Y | NA | Y | Y | NA | Y | NA | 5 |
| Barghouthi Achalu and Mensa | Y | Y | Y | Y | NA | Y | Y | Y | 7 |
| Agalu and Mekonnen, | Y | Y | Y | Y | NA | Y | Y | Y | 7 |
| Gerina et al | Y | Y | Y | Y | Y | Y | Y | NA | 7 |
| Garedow et al., 2022 | Y | Y | NA | Y | Y | U | Y | Y | 6.5 |
| La Vecchia et al., | Y | Y | Y | Y | Y | U | Y | Y | 7.5 |
| Anteneh et al., | Y | Y | Y | Y | Y | Y | NA | Y | 7 |
| Fentie et al., | Y | Y | Y | Y | NA | U | Y | Y | 6.5 |

**Key:** **Y**= Yes (1); U= Unclear (0.5); **NA**= No (0)

**Question codes:**

1. Were the criteria for inclusion in the sample clearly defined?
2. Were the study subjects and the setting described in detail?
3. Was the exposure measured in a valid and reliable way?
4. Were objective, standard criteria used for measurement of the condition?
5. Were confounding factors identified?
6. Were strategies to deal with confounding factors stated?
7. Were the outcomes measured in a valid and reliable way?
8. Was appropriate statistical analysis used?
